# Reimagining pheromone signalling in the model nematode *Caenorhabditis elegans*

**DOI:** 10.1371/journal.pgen.1007046

**Published:** 2017-11-02

**Authors:** Mark Viney, Simon Harvey

**Affiliations:** 1 School of Biological Sciences, University of Bristol, Bristol, United Kingdom; 2 School of Human and Life Sciences, Canterbury Christ Church University, Canterbury, United Kingdom; University of California San Francisco, UNITED STATES

## Overview

*Caenorhabditis elegans* is an important, widely used developmental and genetic model. A pheromone has long been known to cause juvenile developmental arrest in *C*. *elegans*, a phenomenon that is common among nematodes more widely. Many novel effects of this pheromone are now being discovered—most recently, that exogenous supply of this pheromone controls adult worms reproduction. Here, we suggest that to properly understand and interpret these phenomena, *C*. *elegans* natural ecology must be considered, about which rather little is known. With this perspective, we suggest that *C*. *elegans* pheromone signalling evolves very locally, such that there are different dialects of pheromone signalling among ecological communities and among kin groups, and we also argue that pheromone signals may also evolve to be manipulative and dishonest. New approaches must be undertaken to study these phenomena in *C*. *elegans*. While model systems have been tremendously important tools in modern biological research, taking account of their natural history is necessary, and key, to properly understand and interpret laboratory-based discoveries.

## Introduction

Dobzhansky’s dictum—that nothing in biology makes sense except in the light of evolution—has served biology well. But we would add to this that nothing observed of an organism in a laboratory makes sense except in the light of that organism’s natural history. This may not be a controversial statement—indeed, such calls have been made before [1–3]—but it is particularly apposite to a recent spurt of discoveries in the nematode worm *C*. *elegans* that, we suggest, require a critical reinterpretation in the light of the worm’s natural history. Specifically, new work is uncovering how *C*. *elegans* worms communicate with each other using a pheromone to affect each other’s reproduction [4]. Here, we suggest that properly understanding and interpreting these results requires an ecological perspective and that we therefore need to reevaluate how we study these phenomena in this important model organism. Beyond *C*. *elegans*, studies of other nematodes—the animal parasitic nematode *Strongyloides ratti* [5, 6] and the beetle-associated *Pristionchus pacificus* [7]—have considered these species’ ecology in interpreting laboratory-based results; it’s now time for the *C*. *elegans* field to do this too.

A few model systems—*Escherichia coli*, yeast, the fly, the worm, and the mouse—have been tremendously important in discovering and understanding a wide range of biological phenomena. Work with *C*. *elegans* has made major contributions to understanding animal development and discovering the control of behaviour, to mention just 2 areas. The research success of model systems is largely because they have been studied in tightly controlled laboratory environments. But correctly interpreting results obtained from laboratory study of lab-adapted organisms can potentially miss important, different interpretations and alternative explanations if the organism’s natural history is not considered.

## C. elegans

*C*. *elegans* is a free-living nematode. In its 3-day life cycle, offspring of adult worms moult through 4 larval stages (L1–L4) before moulting back into the adult stage. This life cycle is shared by most nematodes, including parasitic species, which are of enormous medical and agricultural importance. For *C*. *elegans*, its life cycle contains a development choice, in which an L3 stage can either continue (via an L4), growing into a reproductive adult, or can instead arrest development as an alternative L3 stage called a dauer larva. Dauer larvae have a specialised structure, physiology, and behaviour that allow them to persist in the environment for weeks. When environmental conditions improve, dauer larvae resume development, growing (via the L4 stage) into reproductive adults.

A larva’s choice of whether or not to arrest development as a dauer larva depends on its environment; specifically, temperature (higher temperature favouring dauer arrest), the quantity of bacterial food available (less food favouring arrest), and the quantity of a pheromone in the environment (more pheromone favouring arrest). This pheromone is secreted by all worms, so its environmental concentration—and worms’ sensation of it—is thought to be a way to measure conspecific population density.

In the laboratory, the *C*. *elegans* life cycle is typically nonarrested, with continuous generations of adult, L1, L2, L3, L4, adult; the arrested dauer larva life cycle (adult, L1, L2, dauer, L4, adult) is only rather rarely used, mainly when wanting to study dauer larvae themselves. In the wild, the dauer life cycle likely usually predominates [8–10].

## *C*. *elegans* pheromone

*C*. *elegans* pheromone plays a critical role in worms deciding whether or not to enter dauer larval arrest. It consists of a complex mixture of (apparently nematode-specific [11]) ascaroside molecules—ascarylose sugars attached to various side chains—but also includes some structural variants (such as indole ascarosides). There is also recent evidence of yet other types of molecules too [4, 12, 13]. This suite of ascaroside molecules is a modular library, with the module components deriving from peroxisomal β-oxidation of fatty acids, carbohydrate metabolism, and amino acid catabolism [12, 14]. All told, some 150 different ascaroside molecules have so far been identified in *C*. *elegans* pheromone (with some 200 known from over 20 different nematode species more generally) [11, 12], thereby revealing this pheromone’s substantial complexity.

But *C*. *elegans* pheromone is not just one standard, fixed mixture of molecules. Its composition varies among life cycle stages [15], between sexes [16], and among different worm strains [17, 18]. Further still, the pheromone’s composition is also affected by the worm’s diet [15], and nutritional [12, 19] and metabolic status [20]. Male worms grown in high population density also appear to have an altered production of ascarosides [16], presumably driven by the increased interactions among individual worms in these dense populations. Equally, not all *C*. *elegans* strains are the same in their responses to pheromone signals. There are strain-specific dauer larva formation and foraging behaviour responses to chemically synthesised ascaroside molecules or natural pheromone mixtures [17, 18]. Analogous findings have also been made with the nematode *P*. *pacificus* too [7, 21]. In *C*. *elegans*, genetic variation in genes coding for chemoreceptor molecules at least in part underlies the diversity of pheromone-dependent foraging behaviour phenotypes [22].

Collectively, these results show that *C*. *elegans* pheromone is a complex molecular mixture, whose composition alters depending on very many aspects of a worm’s state and that there are strain-, state-, and situation-specific responses to pheromone. This suggests that the pheromone is a very information-rich signal with which worms communicate.

## Novel roles for *C*. *elegans* pheromone

The original role ascribed to *C*. *elegans* pheromone was inducing the development of dauer larvae, but now an increasingly diverse set of roles have been (and continue to be) discovered for it. For example, pheromone acts as a chemoattractant between adult males and adult hermaphrodite worms [13, 20], and it promotes aggregation of worms [10] and foraging behaviour [18]. Exogenously supplied pheromone can also increase adult lifespan and fecundity and accelerate hermaphrodite development and the maintenance of their germline precursor cells [4, 23–25]. Worms’ response to pheromone is also condition dependent, with the progeny of poorly fed mothers less likely to develop into dauer larvae in response to exogenous pheromone [26]. Collectively, these results are therefore revealing how *C*. *elegans* pheromone can affect multiple (perhaps all) life cycle stages and that it can also have major effects on adult reproduction. Moreover, these effects will all act to change the population dynamics of *C*. *elegans*.

## *C*. *elegans* ecology

Our understanding of *C*. *elegans* natural ecology is embarrassingly slim [27], especially in comparison with the exquisite detail with which we understand elements of its anatomy, cell biology, and genetics. It lives in ephemeral habitats, principally rotting vegetation (though it is also found on molluscs and arthropods [28–32]), where there are likely extended periods when there is no food and then relatively short-lived periods of abundant food. The dauer stage allows worms to persist in food-depleted environments; that the dauer stage is very commonly found in the environment [8–10] is testament to the frequency with which there is insufficient food available. When food is available, adults rapidly exploit it to reproduce. Wild populations are probably relatively small, with dauer larvae being common in populations of tens of thousands of worms (which could be due to only a few generations of reproduction), suggesting that this may be the maximum size to which a local population can grow [33, 34]. *C*. *elegans* shows significant population genetic differentiation among sample locations [35], with the abundance of different genotypes slowly changing over time [36].

For *C*. *elegans* in the wild, the 2 most critical components of its fitness are (i) larvae deciding whether or not to undergo dauer arrest and (ii) post-dauer larva adult reproduction. If the first of these decisions is wrongly made, then one could be a dauer larva when food is available or be an adult trying to grow and reproduce when there’s no food. Analogously, wrong decisions concerning adult reproduction could also be catastrophic. Pheromone affects (and perhaps drives) both of these major components. This is a remarkable conclusion because it means that extraneous chemical signals are significantly, directly affecting components of worm fitness, which begs the question of why.

## *C*. *elegans* pheromone signalling in an ecological setting—An alternative view

We suggest 3 different, but interrelated, hypotheses concerning *C*. *elegans* pheromone signalling in the wild.

### 1. Pheromone signalling will differ among different ecological communities

Small populations of worms (perhaps numbering a few thousand at most) will interact via the pheromone, which likely acts over short physical distances (perhaps a few centimetres at most) within small-sized patches of food where growing worm populations are found [37]. Pheromone signalling will therefore evolve in these settings, where there will be repeated interactions among members of the ecological community. If communities are discrete from each other, then there is the potential for the evolution of community-specific dialects of pheromone signalling.

Here, the underlying hypothesis is that *C*. *elegans* pheromone is not a common, species-wide signal [4, 24]. While the molecular components of the pheromone are common across the species, we hypothesise that (i) pheromone composition will differ among strains and ecological communities and (ii) phenotypic responses to pheromone signals will differ among strains and communities. To use the analogy of language, while all individuals might use the same basic language, different subgroups develop dialects, and so come to ascribe different meanings to the same word, and may even have subgroup-specific words. For *C*. *elegans* pheromone, ascarosides modularity [12] and the high diversity of the associated chemoreceptors [22] might particularly facilitate the rapid evolution of pheromone signalling and its diversification among ecological communities.

This view is supported by the observation of strain-specific production of ascarosides and strain-specific phenotypic responses to pheromone [17]. Our current understanding of the population genetics of *C*. *elegans* is also consistent with this scenario [35, 36], but much more work needs to be done in this regard. However, more fully testing the hypothesis of community-specific signalling will require laboratory study of multiple strains obtained from single ecological communities and even studying natural communities of worms in the wild. We also need to understand what an ecological community is, its stability, and migration among communities. It is also of note that *C*. *elegans* is routinely found with other *Caenorhabditis* species [27], and pheromones may also be involved in interspecific effects here. We also need to investigate how the pheromone’s molecular components act and interact in signalling and worms’ chemoreception of this. For example, is it the absolute concentration of different individual molecules or is it molecular ratios between different pheromone molecules that are key to pheromone signalling? Studies with the nematode *P*. *pacificus* show asymmetrical responses to pheromone among different strains [7, 21, 38], which is consistent with this idea of community-specific signalling, suggesting that such phenomena may occur among nematodes more widely.

### 2. Pheromone signalling can be used for private signalling among kin groups

Evolutionary success comes from maximising inclusive fitness—one’s own individual fitness and that of close relatives. It follows from this that signalling systems that allow kin to communicate to facilitate inclusive fitness can be selected for [39]. A few *C*. *elegans* likely colonise a newly available food patch, which would lead to kin groups in natural *C*. *elegans* communities, and this might drive the evolution of kin-specific pheromone signalling. Here, the underlying hypothesis is a specific version of 1 (above), in which kin groups would have their own pheromone dialect.

While these pheromone signals are publically broadcast into the environment, correct interpretation of them could be specific to an ecological community or kin group. In support of this idea, game theory studies have shown that public signals can evolve [40].

Consistent with this idea of within–kin-group signalling are the findings that pheromone signals can depend on worm state [12, 15, 19, 20] because this type of information might be key information to be shared via pheromone signals to maximise inclusive fitness. Rather little is known about the population genetics of *C*. *elegans*, particularly at small, local, ecologically relevant scales [36]. Understanding this, including whether or not kin groups exist and persist, is therefore a research priority.

### 3. Pheromone signalling can be dishonest

All animals compete—directly or indirectly—for limiting resources such as food, mates, etc. Dishonest pheromone signalling could be used by *C*. *elegans* for indirect competition so that the dishonest signal-sender succeeds at the signal recipient’s expense. Because pheromone controls both larval arrest and adult reproduction, a dishonest signalling worm could enhance its own success by manipulating others’ reproduction or larval arrest to the dishonest signaller’s advantage. For example, consider a dishonest signal that caused other worms to arrest as dauer larvae even when there was food available. This would allow the dishonest signal-sender to exploit the food themselves. The cocktail party analogy is calling through to the next room, “There’s no need to come through to the buffet, there’s nothing left,” all the while stuffing one's face with smoked salmon.

Theory predicts—and this is empirically supported—that honest signals are sent in 2 settings [39]. Firstly, to kin rather than nonkin because the fitness of kin is a component of an individual’s own fitness, and so honest signalling to kin maximises the signal-sender's inclusive fitness [39, 41, 42]. Secondly, to individuals with whom one repeatedly interacts because a dishonest signal only has value to the signal-sender if the signal-receiver acts on the signal as if it were honest [42, 43]. Put simply (and anthropomorphically), if you’re repeatedly caught lying, then you can’t continue to lie and be believed. Think back to the cocktail party buffet.

There is evidence suggestive of the existence of dishonest signalling in *C*. *elegans*; specifically, there is a worm strain whose pheromone strongly induces dauer larva formation in other strains but not in itself [17], with potentially analogous results in the nematode *P*. *pacificus* [7, 21, 38]. The evolution of dishonest signalling critically depends on the nature and frequency with which individuals interact. Therefore, as above, understanding the membership and dynamics of *C*. *elegans* communities and migration among communities is necessary to fully investigate the existence of dishonesty in *C*. *elegans* pheromone signalling.

## Summary and prospects

In summary, we suggest that *C*. *elegans* pheromone signalling acts and evolves in ecological communities, including kin groups, which may drive the development of community-specific pheromone signalling, including private among-kin signalling as well as signalling that is not always honest.

Current approaches to studying pheromone signalling usually use a single strain of worm (very often the laboratory strain N2), and when multiple strains are used, they are often from disparate geographical sites: these experimental approaches can’t address the alternative hypotheses we have proposed here. Instead, we suggest that pheromone signalling needs to be studied in groups of strains, ideally from true ecological communities. We also need to understand much more about the natural ecological community of *C*. *elegans*, including how genetic diversity is structured at smaller scales, whether or not kin groups exist within these communities, and the extent of migration among communities. We have no idea of the spatial and temporal reach of pheromone signals in the natural environment, and understanding this (which will be hard) will be key in defining ecological communities.

While model systems have been enormously important tools in discovering a myriad of biological phenomena, as more complex phenomena are investigated, including how organisms interact with each other and with their environment, then a good understanding of these models’ natural history is required. In this way, laboratory-based research and field-based ecology will come together to make the next major advances in biology.
